# A step forward understanding HIV-1 diversity

**DOI:** 10.1186/s12977-016-0259-8

**Published:** 2016-04-19

**Authors:** Redmond P. Smyth, Matteo Negroni

**Affiliations:** Architecture et Réactivité de l’ARN, Institut de Biologie Moléculaire et Cellulaire du Centre National de la Recherche Scientifique, Université de Strasbourg, Strasbourg, France

**Keywords:** Human immunodeficiency virus, Genetic diversity, Host restriction factors, Hypermutations, Recombination

## Abstract

Human immunodeficiency virus (HIV) populations are characterized by extensive genetic diversity. Antigenic diversification is essential for escape from immune selection and therapy, and remains one of the major obstacles for the development of an efficient vaccine strategy. Even if intensive efforts have been made for understanding the molecular mechanisms responsible for genetic diversity in HIV, conclusive data in vivo is still lacking. Recent works have addressed this issue, focusing on the identification of the sources of genetic diversity during in vivo infections and on the estimate of the pervasiveness of genetic recombination during replication in vivo. Surprisingly, it appears that despite the error-prone nature of the viral polymerase, the bulk of mutations found in patients are indeed due to the effect of a cellular restriction factor. This factor tends to hypermutate the viral genome abolishing viral infectivity. When hypermutation is incomplete, the virus retains infectivity and converts the effect of the cellular factor to its advantage by exploiting it to generate genetic diversity that is beneficial for viral propagation. This view contrasts the long-standing dogma that viral diversity is due to the intrinsic error-prone nature of the viral replication cycle. Besides hypermutations and mutations, recombination is also a pervasive source of genetic diversity. The estimate of the frequency at which this process takes place in vivo has remained elusive, despite extensive efforts in this sense. Now, using single genome amplification, and starting from publically available datasets, it has been obtained a confirmation of the estimates previously made using tissue culture studies. These recent findings are presented here and their implications for the development of future researches are discussed.

## Background

Viral genetic diversity is essential for escape from immune selection and therapy, and remains one of the major obstacles to the eradication of the immunodeficiency virus (HIV). Although the mechanisms of HIV genetic diversification are well understood in vitro and in cell culture models, in vivo data has been lacking. A recent burst of papers lead us one step closer to understanding these mechanisms in vivo. Here we discuss the main findings described in these works that shed new light on the mechanisms fostering HIV genetic diversity in vivo.

## Main text

A large number of studies have measured viral mutation rates in cell culture [1, 2]. These studies have pointed to a critical role of the viral reverse transcriptase (RT) enzyme in generating mutations, leading to the idea that RT is the dominant producer of genetic diversity. Cuevas and colleagues have evaluated the HIV-1 mutation rate in vivo by analyzing the frequency of stop codons found in patient-derived sequences [3]. Their method is based on the assumption that lethal mutations, not being inherited, cannot be selected, thereby providing a snapshot of what is generated by the mechanism of mutation itself. In this way, they were able to directly measure HIV mutation rates from both plasma virus and cells isolated from infected individuals. They found a mutation rate of 9.3 × 10^−5^ mutations per base pair in plasma virus, which is a rate compatible with estimates obtained from cell culture experiments. Interestingly, the HIV mutation rates found in DNA from peripheral blood mononuclear cells was 44 times higher, and around 98 % of the mutations found were generated by the APOBEC family of proteins, and not by the reverse transcriptase. These cellular enzymes hypermutate the reverse transcription product, leading to the insertion of aberrant amino acids and the generation of nonsense mutations incompatible with viral infectivity. Certainly, the large difference in mutation rates found between plasma virus and infected cells evidences strong purifying selection to remove non-infectious hypermutated genomes [4]. Importantly, however, the authors provide evidence that HIV-1 can hijack this cellular defense machinery to foster its sequence diversification. Indeed, they first showed that the activity of the APOBEC family protein A3G does not result in an all-or-nothing pattern in vivo, as it was previously thought, but that the number of mutations found in each sequence spans across two orders of magnitude. Next, they demonstrated that mutation rates varied according to the clinical status of the patient, and that mutation rates and disease progression were correlated. They saw a lower overall mutation rate in rapid progressors compared to normal progressors and a negative correlation between set point viral load and mutation rate. This is consistent with a strong antiviral activity by APOBEC. However, when the authors stratified their data by the amount of editing (no editing, low-level editing, or high editing), sequences isolated from rapid progressors contained a higher number of low-level editing events when compared to normal progressors. Furthermore, when looking at these sequences with low-level editing, the authors found a positive correlation between set point viral load and mutation rate. Thus, it appears that the virus can harness low levels of A3G activity to promote beneficial genetic diversity, whereas higher levels of editing lead to slower disease progression. This is good and bad news for the development of Vif-APOBEC inhibitors as it shows that inhibition of Vif can push HIV beyond the limit of mutations that it is able to tolerate, but also that inefficient inhibition can instead promote viral pathogenesis.

Presumably, even low level editing—defined by the authors as 1–10 “lethal” mutations per sequencing library—have the potential to completely block the viral replication. However, in situations where cells are co-infected with multiple viruses, complementation of defective proteins may allow the recovery of viable virus. Furthermore, genomes from different viruses may be co-packaged into the same viral particle and “repaired” during the next replication cycle. This occurs via retroviral recombination during reverse transcription, when the RT switches template between the two co-packaged genomes, generating a chimerical DNA molecule. Although recombination has been intensively studied in cell culture and in vitro, it has been difficult to quantify its contribution to viral genetic diversity in vivo due to the difficulty of detecting and tracking the history of individual viruses. Cromer and colleagues tackled this problem using data obtained from single genome amplification at early time points [5]. Previous studies have shown that just after infection, viral diversity is sufficiently low to reconstruct the genetic makeup of the founder virus [6]. Using publically available datasets, Cromer and colleagues were able to identify several individuals infected with multiple viruses at the point of transmission. They then were able to quantify the number of recombination events occurring by identifying sequences that were a mix of the two founder viruses. Interestingly, they show that HIV recombines on average 5-14 times per genome per replication cycle, validating observations from cell culture studies [7]. In the light of the pervasive mutagenesis introduced by APOBEC, it is likely that recombination is required for shuffling APOBEC induced mutations within the viral population.

## Conclusions

Many are the questions that remain open in the field. (1) Do hypermutated non-infectious genomes serve as reservoirs for rescuing mutations by superinfecting viruses through recombination, extending the sequence space explored by the virus? (2) What is the relevance of the differences in the level of expression of A3G in the different cell types for sequence diversification at the level of transmission? The limited number of viruses transmitted during primary infections forces the viral population through a bottleneck from which genetic diversification must then be re-established. Do the various cell types infected during spreading in the organism introduce sequence diversification at different paces depending on the stage and tissue infected? (3) What is the profile of APOBEC proteins expression in elite-controllers and long-term non-progressors? Do some individuals show differences in HIV mutation rates? Are differences more related to host genetic or to the viral genotype? (4) Are other nucleic acids editing enzymes involved in controlling genetic diversity? (5) What is the implication of the genome in modulating sequence diversity? Another recent work [8], indicates that the frequency of generation of sequence divergence is not homogeneous along the HIV-1 *env* gene. The authors reported that the genomic structure (both primary and secondary) of the regions encoding for the most external portions of the surface subunit gp120 is less prone to the insertion of mutations, both by the viral polymerase and by APOBEC proteins. This caveat would limit the insertion of mutations in regions with a level of genetic diversity close to the upper limit tolerable for viability.

Altogether these observations tell us that viruses can divert weapons used against them by modulating their effect for its own purposes. Our understanding of the arms race between viruses and their hosts is manifestly far from being complete.
